# Research on Yaw Stability Control Strategy for Distributed Drive Electric Trucks

**DOI:** 10.3390/s23167222

**Published:** 2023-08-17

**Authors:** Feng Gao, Fengkui Zhao, Yong Zhang

**Affiliations:** College of Automobile and Traffic Engineering, Nanjing Forestry University, Nanjing 210037, China; gaofeng@njfu.edu.cn (F.G.); zfk@njfu.edu.cn (F.Z.)

**Keywords:** distributed drive, electric trucks, layered control, linear quadratic regulator

## Abstract

With the advancement of vehicle electrification and intelligence, distributed drive electric trucks have emerged as the preferred choice for heavy-duty electric trucks. However, the control of yaw stability remains a significant issue. To tackle this concern, this study introduces a layered control strategy for yaw moment. Specifically, the upper layer utilizes a yaw moment controller based on linear quadratic regulator (LQR) to compute the additional yaw moment required. Additionally, in order to enhance the performance of the yaw moment controller, the weight matrix in LQR is optimized using a hybrid Genetic Algorithm and Particle Swarm Optimization algorithm (GA-PSO). The lower layer consists of a torque distribution layer, which establishes an objective function for minimizing tire utilization rate. Quadratic Programming algorithm is then employed to compute the optimal torque distribution value, thereby improving the vehicle’s stability. Subsequently, the stability control effects of the vehicle are simulated and compared on the Matlab/Simulink Trucksim joint simulation platform using four control strategies: the proposed control strategy, SMC, LQR, and without yaw moment control. These simulations are conducted under two working conditions: serpentine and double lane change. The results demonstrate that the proposed approach reduces the average yaw rate by 14.4%, 19.6%, and 42.15% while optimizing the average sideslip angle by 25.9%, 24.8%, and 52.3% in comparison to the other three control strategies. Consequently, the proposed control strategy significantly enhances the driving stability of the vehicle. Furthermore, the optimized allocation method reduces the average tire utilization rate by 42.6% in contrast to the average allocation method, thereby improving the stability control margin of the vehicle. These findings successfully validate the efficiency of the yaw stability control strategy presented in this article.

## 1. Introduction

In recent years, there has been a growing demand for electric trucks in the heavy-duty truck industry due to increasing environmental requirements and restrictions on traditional fuel vehicles. The power system layout of electric trucks can be classified into two categories: centralized and distributed [1]. Traditional centralized drive electric trucks typically utilize a single motor to control the movement of the entire vehicle. On the other hand, distributed hub motor-driven electric trucks mount the drive motor on the wheel rim, directly powering the wheels. This design enables independent control of the driving torque for each wheel, resulting in higher response accuracy and faster power system response speed [2,3,4]. Through the installation of motors on each wheel, this distributed solution significantly increases the unsprung mass of the entire vehicle. As a result of this increase in unsprung mass, the vehicle may encounter more pronounced bumps and vibrations when driving over uneven road surfaces, thus compromising ride comfort. Moreover, the augmented unsprung mass can negatively influence the vehicle’s road grip, potentially resulting in reduced handling performance. By strategically arranging the output torque of the hub motor, an external yaw torque can be generated and applied to yaw motion control, thereby enhancing the stability of the vehicle. Consequently, the development of appropriate yaw stability control strategies has become a current research focus.

Due to the distributed control of the electric drive system, the controllability of the vehicle chassis is significantly improved. Currently, the most commonly used method for active stability control of distributed electric drive vehicles is direct yaw moment control. Many scholars have extensively researched direct yaw torque control of distributed drive electric vehicles. Generally, this control method adopts a hierarchical strategy. The upper controller tracks the reference value of the vehicle’s motion state and outputs the target additional yaw torque. The lower controller then allocates and controls the torque of each wheel hub motor based on the additional yaw torque provided by the upper controller.

Various control theories have been applied to the stability control of distributed drive electric vehicles, including the proportional integral differential PID [5,6], linear quadratic regulator (LQR) [7,8], fuzzy control [9,10], sliding mode control (SMC) [11,12], and model predictive control (MPC) [13,14,15]. Researchers have utilized these control theories for direct yaw torque control. For instance, Ahmed et al. [16] proposed a yaw torque control algorithm based on a PID controller, which effectively adjusted the vehicle’s yaw rate. Ge et al. [17] introduced a direct yaw torque controller based on an adaptive sliding mode algorithm that demonstrated good control effects on yaw rate and sideslip angle, improving vehicle adaptive stability control. Ma et al. [18] presented a control algorithm based on fuzzy logic to enhance the linear stability of wheel motor four-wheel drive vehicles. In the upper controller, an NMPC controller was proposed to calculate the required tire slip rate as a virtual control input [19]. In the lower layer controller, a linear MPC (LMPC) controller was suggested, which allocated virtual control input to motor torque, thereby improving the stability of distributed drive electric vehicles under extreme conditions. However, PID controllers lack robustness and demonstrate poor adaptability and control effectiveness in complex conditions. Sliding mode control suffers from chattering problems and complex models. The design of fuzzy controllers lacks systematicity and requires researchers to possess extensive experience in debugging fuzzy rules. Meanwhile, when model accuracy is low, MPC controllers may yield inaccurate predictions and high computational complexity. Considering the stability control requirements of distributed drive electric trucks, LQR control stands out due to its strong robustness, small steady-state error, and simple implementation methods. Therefore, this article employs the LQR algorithm to design a layered control strategy for direct yaw torque control.

Although the LQR is widely employed in vehicle control, there remains an issue concerning empirical parameter conditions. Therefore, in order to effectively control serpentine oscillation, Lei et al. [20] used the Particle Swarm Optimization (PSO) algorithm to optimize LQR parameters. Wang et al. [21] proposed a gain scheduling robust linear-quadratic regulator (RLQR) that addressed the limitations of parameter uncertainty by adding an additional control term to the feedback contribution of the conventional LQR. Xie et al. [22] introduced an enhanced LQR based on adjusted weight coefficients, employing fuzzy rules to modify the weight coefficients of the LQR and enhance the performance of the vehicle’s direct yaw torque control system. Experiments conducted in [23] demonstrated that utilizing a Genetic Algorithm (GA) to determine the parameters of the LQR resulted in better control effects compared to those obtained through the trial and error method. Although the aforementioned research has partially optimized the parameters of the LQR, further optimization is still necessary when applying it to the stability control of distributed electric trucks.

Therefore, this article focuses on distributed drive electric trucks and proposes a layered control strategy for direct yaw torque. The main contributions of this article are as follows:

(1) A linearized reference model for distributed drive vehicles is established to calculate the necessary state variables for control. Additionally, an upper layer yaw moment controller is designed using the LQR method;

(2) The weight matrix in the LQR yaw torque controller is optimized using a combination of the Particle Swarm Optimization algorithm and Genetic Algorithm (GA-PSO), which enhances the control effect;

(3) A lower layer torque distributor is developed based on the principle of minimum tire utilization. The optimal torque distribution value is calculated using the Quadratic Programming algorithm to improve vehicle stability;

(4) The Matlab/Simulink Trucksim joint simulation platform is employed in this study to simulate and compare the effects of vehicle stability control under four different control strategies, considering two operating conditions: serpentine and double lane change.

The rest of this article is organized as follows: the second part establishes a two-degree-of-freedom vehicle dynamics reference model, the third part presents a yaw stability control strategy, and the fourth part conducts experiments and analysis. Finally, the fifth part summarizes the contribution of this article and proposes future research directions.

## 2. Vehicle Model

### 2.1. Vehicle 2DOF Reference Mode

**Figure 1:**
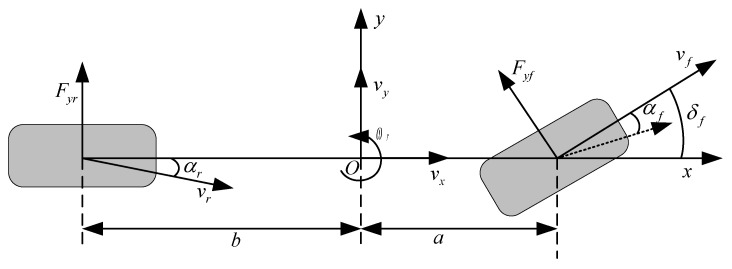
Vehicle 2DOF model.

By studying the kinematic and dynamic characteristics of a vehicle, we can determine the criteria to measure its stability. These criteria include the sideslip angle and the yaw rate, which reflect the degree of deviation and steering characteristics when the vehicle turns. To design a controller for lateral stability, a two-degree-of-freedom model (2DOF) is commonly used to describe the expected state of vehicle yaw and lateral motion. This model is simple, practical, and has fewer parameters. Specifically, the model assumes that the vehicle body is rigid, treating the left and right wheel angles and sideslip angles as equal. It only considers the vehicle’s yaw and lateral motion, ignoring vertical and pitch motion, and assumes constant longitudinal velocity. The model also assumes ideal adhesion conditions and does not account for the effects of vehicle suspension and changes in vertical load on tire sideslip characteristics. Figure 1 depicts the vehicle’s two-degree-of-freedom model, whereas Equations (1) and (2) establish the vehicle model equations.

(1) $$mv_{x}(\omega_{r}+\dot{\beta })=F_{yf}\cos\delta_{f}+F_{yr}$$

(2) $$I_{z}{\dot{\omega }}_{r}=aF_{yf}\cos\delta_{f}-bF_{yr}+\Delta M_{z}$$

where $v_{x}$ is the longitudinal speed of the vehicle; $\omega_{r}$ is the yaw rate; β is the sideslip angle; $F_{yf}$ and $F_{yr}$, respectively, represent the total lateral force of the front and rear tires; m is the mass of the entire vehicle; $I_{z}$ is the moment of inertia about the z-axis; a and b are the distances from the center of mass of the vehicle to the front and rear axles, respectively; $\delta_{f}$ is the front wheel angle; and $\Delta M_{z}$ is the additional yaw moment.

Our research mainly focuses on the driving conditions of vehicles under low lateral acceleration conditions. In this case, the behavior of the tire can be modeled and analyzed using a linear model with good approximation. Compared to magic formula models, linear models have simpler mathematical forms, fewer parameter requirements, and computational complexities. This enables us to simulate and analyze more quickly and achieve reliable results with limited computing resources. So, we assume that the tire is in a linear region, and the lateral angle of the tire is proportional to the lateral force it bears, as shown in Equation (3).

(3) $$\left\{\begin{array}{l} F_{yf}=C_{f}\alpha_{f}\\ F_{yr}=C_{r}\alpha_{r} \end{array}\right.$$

where $C_{f}$ and $C_{r}$ are the cornering stiffnesses of the front and rear axles, respectively; and $\alpha_{f}$ and $\alpha_{r}$ are the slip angles of the front and rear tires, respectively.

The tire sideslip angle can be calculated from Equation (4).

(4) $$\left\{\begin{array}{l} \alpha_{f}=\beta +\frac{a\omega_{r}}{v_{x}}-\delta_{f}\\ \alpha_{r}=\beta -\frac{b\omega_{r}}{v_{x}} \end{array}\right.$$

Assuming small front wheel angle, $\cos\delta_{f}\approx 1$, the state space Equation (5) can be obtained by synthesizing Equations (1)–(4) [24].

(5) $$\dot{X}=AX+Bu+E\delta_{f}$$

where

(6) $$X={\left[\begin{array}{cc} \beta & \omega_{r} \end{array}\right]}^{T}$$

(7) $$A=\left[\begin{array}{cc} -\frac{\left(C_{f}+C_{r}\right)}{mv_{x}} & -\frac{aC_{f}-bC_{r}}{mv_{x}^{2}}-1\\ -\frac{aC_{f}-bC_{r}}{I_{z}} & -\frac{a^{2}C_{f}+b^{2}C_{r}}{I_{z}v_{x}} \end{array}\right]$$

(8) $$B={\left[\begin{array}{cc} 0 & \frac{1}{I_{z}} \end{array}\right]}^{T}$$

(9) $$E={\left[\begin{array}{cc} \frac{C_{f}}{mv_{x}} & \frac{aC_{f}}{I_{z}} \end{array}\right]}^{T}$$

(10) $$u=\Delta M_{z}$$

### 2.2. Calculation of Reference Values for State Variables

To ensure vehicle stability, it is crucial to maintain the yaw rate and sideslip angle at ideal levels [25]. By employing the 2-degree-of-freedom linear models (1) and (2), the yaw rate and the sideslip angle of the vehicle in a steady state at a constant speed can be calculated [26]. Equation (11) used for steady-state value calculation completely ignores the existence of torque vectors and their abilities to alter the vehicle’s steady-state response.

(11) $$\left\{\begin{array}{l} \omega_{st}=\frac{v_{x}}{L\left(1+Kv_{x}^{2}\right)}\delta_{f}\\ \beta_{st}=\left[\frac{b}{L\left(1+Kv_{x}^{2}\right)}+\frac{mav_{x}^{2}}{C_{r}L^{2}\left(1+Kv_{x}^{2}\right)}\right]\delta_{f} \end{array}\right.,\;\begin{array}{c} K=\frac{m}{L^{2}}\left(\frac{a}{C_{r}}-\frac{b}{C_{f}}\right) \end{array}$$

where $\omega_{st}$ is the steady-state yaw rate; $\beta_{st}$ is the steady-state sideslip angle; K is a stability factor; and *L* is the vehicle wheelbase.

The boundary values of sideslip angle and yaw rate are related to the road adhesion coefficient, according to references [27,28]. Considering the limitations of road adhesion coefficient, the final reference value of yaw rate and sideslip angle are

(12) $$\begin{array}{c} \omega_{d}=\left\{\begin{array}{ll} \omega_{st}, & \left|\omega_{st}\right|<\frac{0.85\;\mu g}{v_{x}}\\ \frac{0.85\;\mu g}{v_{x}}\mathrm{sign}\left(\omega_{st}\right), & \left|\omega_{st}\right|\geq \frac{0.85\;\mu g}{v_{x}} \end{array}\right. \end{array},\;\beta_{d}=\left\{\begin{array}{ll} \beta_{st}, & \left|\beta_{st}\right|<\beta_{\max}\\ \beta_{\max}\mathrm{sign}\left(\beta_{st}\right), & \left|\beta_{st}\right|\geq \beta_{\max} \end{array}\right.$$

where $\omega_{d}$ is the reference value of yaw rate; $\beta_{d}$ is the reference sideslip angle; $\beta_{\max}=\arctan(0.02\;\mu g)$ μ is the tire-road friction coefficient; and g is the acceleration of gravity.

## 3. Yaw Stability Control Strategy

**Figure 2:**
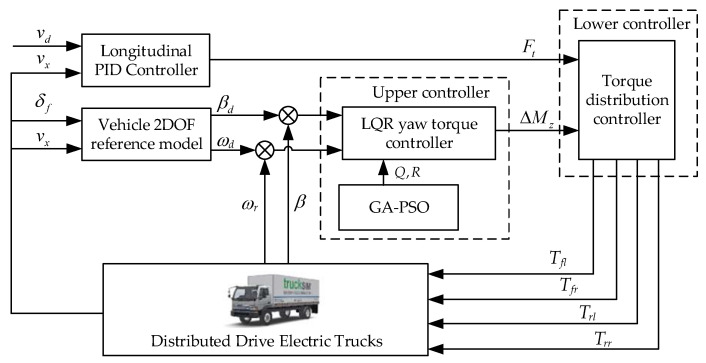
Layered control system framework.

Layered control is one of the best ways to control vehicle yaw stability, which can divide the system structure into multiple small components to improve processing speed, stability, and adaptability. The control system framework designed based on hierarchical control concept is shown in Figure 2. The system consists of five modules: a longitudinal PID controller, a vehicle 2DOF reference model, an upper controller, a lower torque distribution controller, and a distributed drive electric truck as the controlled object. The relevant parameters of the 2DOF reference model can be determined using fitting or optimization techniques by comparing the data between the Trucksim model and the selected monorail model to determine the equivalent parameters of the monorail model.

### 3.1. Design of Longitudinal Speed Controller

The main purpose of the longitudinal speed controller is to track the desired longitudinal speed while also determining the total longitudinal driving force. The longitudinal driver model is controlled by a PID controller illustrated in Equation (13). It calculates the total driving force $F_{t\;}$ of the vehicle by considering the difference between the actual vehicle speed $v_{x}$ and the desired vehicle speed $v_{d}$.

(13) $$F_{t\;}=K_{p}\left(v_{d}-v_{x}\right)+K_{i}\int \left(v_{d}-v_{x}\right)dt+K_{d}\frac{d\left(v_{d}-v_{x}\right)}{dt}$$

where $K_{p}$, $K_{i}$, and $K_{d}$ are the proportional coefficient, integral coefficient, and differential coefficient of the PID controller, respectively.

### 3.2. Upper Controller

#### 3.2.1. LQR Controller Design

The distributed electric truck yaw stability control system adopts a layered control design, and the upper controller is mainly used to calculate the additional yaw torque. The Linear–quadratic regulator is an excellent linear Quadratic form regulator. Its response speed is very fast. It can respond to the system in a short time. It has high precision control capability and can achieve fine motion control in a large range. Therefore, this article uses the centroid sideslip angle and yaw rate, which represent the stability state of the vehicle, as control variables and designs an additional yaw torque feedback controller using LQR optimal control theory to solve the direct yaw torque applicable to the vehicle. Using the error between the actual values of yaw rate and sideslip angle and the reference values as the state variable e, feedback compensation is used to make the vehicle state approach the target value.

(14) $$e=\left[\begin{array}{c} \Delta \beta \\ \Delta \omega_{r} \end{array}\right]=\left[\begin{array}{c} \beta_{d}-\beta \\ \omega_{d}-\omega_{r} \end{array}\right]$$

By combining Equations (5)–(8), the error state space between the actual and reference values of the sideslip angle and yaw rate can be obtained as shown in Equation (15),

(15) $$\dot{e}=\bar{A}e+\bar{B}u$$

where $\bar{A}=\left[\begin{array}{cc} -\frac{C_{f}+C_{r}}{mv_{x}} & -\frac{aC_{f}-bC_{r}}{mv_{x}^{2}}-1\\ -\frac{aC_{f}-bC_{r}}{I_{z}} & -\frac{a^{2}C_{f}+b^{2}C_{r}}{I_{z}v_{x}} \end{array}\right]$; $\bar{B}={\left[0\;-\frac{1}{I_{z}}\right]}^{T}$; $u=\Delta M_{z}$.

In order to ensure that the Linear–quadratic regulator gets the optimal solution, it is necessary to establish the performance index of the controller. Design optimization objective functions for Equations (16) and (17).

(16) $$\min J=\int_{0}^{\infty }\left(e^{T}Qe+u^{T}Ru\right)dt$$

(17) $$\left\{\begin{array}{l} Q=\left[\begin{array}{cc} q_{1} & 0\\ 0 & q_{2} \end{array}\right]\\ R=\left[q_{3}\right] \end{array}\right.$$

where Q is the weighted matrix of the state error, which represents the degree of attention paid to the control target and is a semi positive definite real number Symmetric matrix; R is the weight matrix of the control quantity, which is a positive definite real Symmetric matrix; $q_{1}$ and $q_{2}$ are the error weight coefficients of the actual and reference values of the sideslip angle and yaw rate, respectively; and $q_{3}$ is the degree of limitation of the additional yaw moment.

According to the LQR control principle, the optimal state feedback gain matrix can be obtained,

(18) $$G=-R^{-1}B^{T}P$$

where P is obtained by solving the Riccati Equation of Equation (19).

(19) $$PA+A^{T}P-PBR^{-1}B^{T}P+Q=0$$

Then, the optimal state feedback control quantity is,

(20) u=−Ge

The derivation process and optimal control law of the LQR algorithm indicate that the effectiveness of control in the LQR controller depends on the selection of parameters in matrices **Q** and **R**. As there is no analytical method for parameter selection, it is necessary to choose parameters based on different control objectives and optimization targets. In other words, different control objectives and indicators require different control effects, so it is important to determine the appropriate parameter selection scheme for different application scenarios.

#### 3.2.2. LQR Based on GA-PSO Optimization

**Figure 3:**
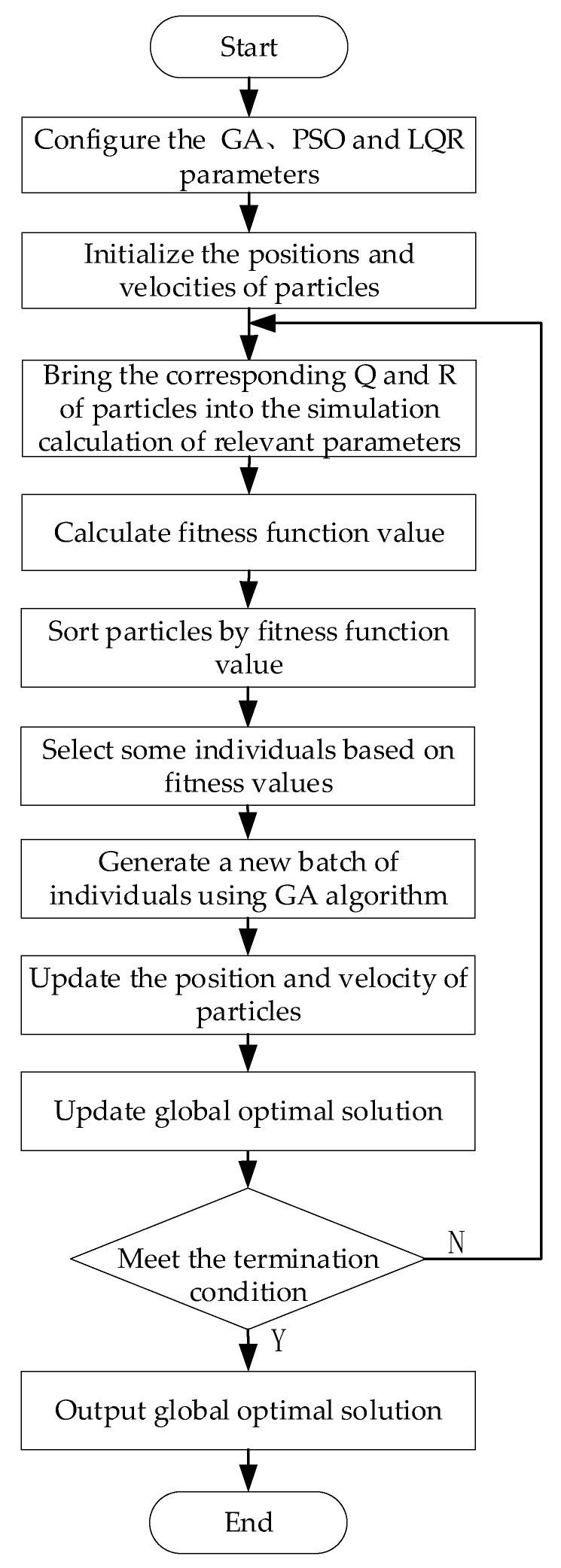
GA-PSO optimized LQR algorithm flowchart.

The GA-PSO algorithm is a combination of the Genetic Algorithm [29] and Particle Swarm Optimization [30]. The Genetic Algorithm is an optimization algorithm that simulates the process of biological evolution to find the optimal solution. On the other hand, Particle Swarm Optimization is an adaptive algorithm that simulates the movement and interaction of particles in the search space to find the optimal solution. The GA-PSO algorithm integrates the crossover and mutation operations from the Genetic Algorithm and incorporates the speed and position update concepts from Particle Swarm Optimization. By combining these two algorithms, we can leverage their respective advantages and better address optimization problems. The algorithm flowchart for applying GA-PSO to optimize the LQR weight matrix is presented Figure 3.

Firstly, the relevant parameters for the algorithm are configured. Next, the initial position and velocity of each particle were randomly generated; we brought the corresponding **Q** and **R** values of each particle into the simulation run; and we calculated the relevant parameters required for the fitness function. Then, we used the fitness function of Equation (21) to calculate their fitness values. The fitness function served as the foundation of particle swarm optimization. In order to enhance the response characteristics of the vehicle, the control objective of vehicle stability considered the relationship between the frequency domain and time domain, as well as the characteristics and working conditions of distributed drive electric trucks. The fitness function was designed based on the integration time and absolute error sum (ITAE) of the sideslip angle and yaw rate.

(21) $$\min F=\int_{0}^{T}t\left|\Delta \beta (t)\right|dt+\int_{0}^{T}t\left|\Delta \omega_{r}(t)\right|dt$$

where *t* refers to the running time.

Particles in the particle swarm were sorted based on their fitness values to identify the optimal particle. Simultaneously, particles with high fitness values were selected, and the GA algorithm was utilized for cross mutation to generate a new set of individuals for these particles. Subsequently, the velocity and position of each particle were updated using the PSO velocity and position update function, as indicated in Equation (22). Moreover, the global optimal solution was updated, and iteration continued until the predefined stopping conditions were satisfied.

(22) $$\left\{\begin{array}{l} V_{i}(k+1)=\lambda V{\left(k\right)}_{i}+c_{1}r_{1}\left(p_{\mathrm{best}\_i\;}-X_{i}(k)\right)+c_{2}r_{2}\left(g_{\mathrm{best}\;}-X_{i}(k)\right)\\ X_{i}(k+1)=X_{i}(k)+V_{i}(k+1) \end{array}\right.$$

where $V_{i}(t)$ and $X_{i}(t)$ are the velocity and position of the ith particle in the kth iteration, $p_{\mathrm{best}\_i\;}$ is the personal optimal solution of the ith particle, $g_{\mathrm{best}\;}$ is the current global optimal solution, $c_{1}$ and $c_{2}$ are learning factors, and $r_{1}$ and $r_{2}$ are random numbers between [0, 1]; λ is the inertial factor.

This article adopts the linear decreasing inertia weight shown in Equation (23),

(23) $$\lambda =\lambda_{start}-(\lambda_{start}-\lambda_{end})\cdot \frac{k}{k_{\max}}$$

where $\lambda_{start}$ is the initial inertia weight; $\lambda_{end}$ is the inertia weight at the maximum number of iterations; k is the current iteration algebra; and $k_{\max}$ is the maximum iterative algebra. Generally speaking, the algorithm performs best with inertia weights $\lambda_{start}=0.9$ and $\lambda_{end}=0.4$. In this way, as the iteration progresses, the inertia weight linearly decreases from 0.9 to 0.4. The larger inertia weight in the initial stage of the iteration maintains the algorithm’s strong global search ability, whereas the smaller inertia weight in the later stage of the selection is conducive to the algorithm’s more accurate local search.

The optimization results corresponding to two different working conditions are shown in Table 1. Condition 1 entails simulating a serpentine maneuver at a speed of 50 km/h on a road surface with a friction coefficient of μ = 0.4. Condition 2 refers to a double lane change simulation conducted at a speed of 80 km/h on a road surface with μ = 0.7.

We will develop a set of switching logic to determine when, how, and why to switch parameter sets in different situations. This may include optimizing results based on specific operating conditions or automatically adjusting control strategies based on changes in vehicle status.

### 3.3. Lower Torque Distributor

After the LQR yaw torque controller optimized by the upper GA-PSO calculates the target additional yaw torque, it is necessary to allocate the driving torque of the four wheels to meet the total driving force and additional yaw torque requirements.

#### 3.3.1. Establishment of Objective Function

The ratio between the road adhesion utilization of a single wheel to its maximum adhesion under current working conditions [31]. It primarily reflects the adhesion utilization between the wheels and the road surface while also characterizing the stability margin of the vehicle. A lower tire utilization rate indicates a larger stability margin, whereas a higher tire utilization rate indicates a smaller stability margin. An increase in the tire utilization rate to 1 indicates that the vehicle is approaching the adhesion limit, which may result in instability. Thus, controlling the tire utilization rate as low as possible is necessary to ensure the driving stability of the vehicle. The objective function for minimizing the utilization of the four wheels and tires can be defined as follows,

(24) $$\min J_{T}=\sum_{i=j}^{4}C_{j}\frac{F_{xj}^{2}+F_{yj}^{2}}{\mu^{2}F_{zj}^{2}},j=fl,fr,rl,rr$$

where $F_{xj}$ is the longitudinal force of the four wheels; $F_{yj}$ is the lateral force of the four wheels; $F_{zj}$ is the vertical load of four wheels; and $C_{j}$ is the weight coefficient of each wheel utilization rate. Due to the fact that the driving motor is not the focus of research in this article, the driving motors of each wheel are considered consistent, with $C_{j}=1$; μ is the tire-road friction coefficient.

For electric trucks with distributed drive, optimizing calculations with the combined effects of longitudinal force, lateral force, and vertical load is complex. According to the friction ellipse, in the extreme case, there is a certain coupling relationship between longitudinal and lateral forces and the wheels. The lateral force of the vehicle cannot be directly controlled for the time being, so only the longitudinal force optimization distribution of the vehicle is considered, which reduces control accuracy and the difficulty in vehicle control. Equation (25) represents the simplified objective function.

(25) $$\min J_{T}=\sum_{i=j}^{4}\frac{F_{xj}^{2}}{\mu^{2}F_{zj}^{2}},j=fl,fr,rl,rr$$

where $F_{xj}$ and $F_{zj}$ are obtained through real-time data output through Trucksim. μ is the adhesion coefficient in the pre-set working condition, which is a constant value.

To simplify the calculation and unify the effective rolling radii of the four wheels, the longitudinal force in Equation (25) is expressed as Equation (26).

(26) $$F_{xj}=\frac{T_{j}}{R},j=fl,fr,rl,rr$$

where $T_{j}$ is the driving torque of each wheel; and R is the tire loaded radius of the four wheels.

The objective function can be expressed as Equation (27).

(27) $$\min J_{T}=\sum_{i=j}^{4}\frac{T_{j}^{2}}{\mu^{2}F_{zj}^{2}},j=fl,fr,rl,rr$$

Convert the objective function into the matrix form shown in Equation (28).

(28) $$\min J_{T}=U^{T}WU$$

where $U={\left[\begin{array}{cccc} T_{fl} & T_{fr} & T_{rl} & T_{rr} \end{array}\right]}^{T}$; $W=\mathrm{diag}{\left[\begin{array}{cccc} \frac{1}{\mu^{2}F_{zfl}^{2}} & \frac{1}{\mu^{2}F_{zfr}^{2}} & \frac{1}{\mu^{2}F_{rl}^{2}} & \frac{1}{\mu^{2}F_{zrr}^{2}} \end{array}\right]}^{T}$.

#### 3.3.2. Restraint Condition

To optimize the allocation of wheel torque, establishing an optimization objective function is necessary while ensuring compliance with longitudinal and lateral control requirements. Each wheel should satisfy both the required additional yaw moment and longitudinal force constraints for the entire vehicle concurrently. The equation representing the additional yaw moment constraint and the equation representing the longitudinal force constraint required by the vehicle are as follows,

(29) $$\left\{\begin{array}{c} \left(F_{xfl}+F_{xfr}\right)\cos\delta_{f}+\left(F_{xrl}+F_{xrr}\right)=F_{t}\\ \frac{d_{f}}{2}\left(F_{xfr}-F_{xfl}\right)\cos\delta_{f}+\frac{d_{r}}{2}\left(F_{xrr}-F_{xrl}\right)=\Delta M_{z} \end{array}\right.$$

where $F_{xfl}$, $F_{xfr}$, $F_{xrl}$, and $F_{xrr}$ are the longitudinal forces of the four wheels; $T_{t}$ is the total torque; $F_{t}$ is the total driving force; and $d_{f}$ and $d_{r}$ represent the track widths of the front and rear axles, respectively.

Considering the constraints provided by Equation (29), in addition, it is important to consider that the torque output of the motor is constrained by its power, and the tire’s contact with the road is limited due to road adhesion conditions. When the torque is positive, it needs to be less than the smaller of the maximum torque and maximum road adhesion. When the torque is negative, it needs to be greater than the largest of the negative values corresponding to the maximum torque and maximum road adhesion. Thus, an inequality constraint for Equation (30) is established to meet the conditions.

(30) $$\max\left(-\mu F_{zj}R,-T_{\max}\right)\leq T_{j}\leq \min\left(\mu F_{zj}R,T_{\max}\right),j=fl,fr,rl,rr$$

where $T_{\max}$ is the maximum torque output by the motor.

#### 3.3.3. Quadratic Programming Optimization Solution

Quadratic Programming refers to a class of optimization problems, whose objective function is a Quadratic function, and the constraints are linear functions. A common algorithm for solving Quadratic Programming problems is an interior point method. After the torque allocation problem is transformed into a Quadratic Programming problem, a more stable torque allocation scheme can be obtained by optimizing the objective function, thus improving the stability and control of the system. Therefore, the torque allocation problem in this paper can be transformed into a Quadratic Programming problem, and the objective function can be expressed as the standard form of Quadratic Programming in Formula (31) by combining the above determined objective function and constraints.

(31) $$\begin{array}{l} \min J_{T}=U^{T}WU\\ s.t.\;\left\{\begin{array}{l} HU=p\\ U_{\min}\leq U\leq U_{\max} \end{array}\right. \end{array}$$

Combining Equations (29) and (30) yields Equations (32) and (33).

(32) $$\left\{\begin{array}{c} W=\mathrm{diag}{\left[\begin{array}{cccc} \frac{1}{\mu^{2}F_{zfl}^{2}} & \frac{1}{\mu^{2}F_{zfr}^{2}} & \frac{1}{\mu^{2}F_{rl}^{2}} & \frac{1}{\mu^{2}F_{zrr}^{2}} \end{array}\right]}^{T}\\ H=\left[\begin{array}{cccc} \cos\delta_{f} & \cos\delta_{f} & 1 & 1\\ -\frac{d_{f}\cos\delta_{f}}{2} & \frac{d_{f}\cos\delta_{f}}{2} & -\frac{d_{r}}{2} & \frac{d_{r}}{2} \end{array}\right]\\ p=\left[\begin{array}{c} T_{t}\\ \Delta M_{z}R \end{array}\right] \end{array}\right.$$

(33) $$\left\{\begin{array}{l} U_{\min}=\max\left(-\mu F_{zj}R,-T_{\max}\right)\\ U_{\max}=\min\left(\mu F_{zj}R,T_{\max}\right) \end{array}\right.,j=fl,fr,rl,rr$$

As the matrix **W** can be either positive definite or semi-positive definite, the torque allocation problem becomes a convex Quadratic Programming problem. Common methods for solving such convex Quadratic Programming problems are the interior point method, Lagrange multiplier method, fixed point method, and efficient set method. Comparatively, the efficient set method provides higher computational efficiency and requires fewer iterations. It can efficiently handle both equality and inequality constraints. The iteration points are solved at the constraint boundary until the optimal solution is found, ensuring that the iteration points always remain within the feasible region, resulting in a significant reduction in the computational workload. Therefore, this paper utilizes the efficient set method, along with relevant MATLAB command statements, to solve the convex Quadratic Programming problem.

Once the optimal solution of the Quadratic Programming problem has been obtained using the aforementioned solution, the resulting torque allocation for each wheel can be determined. This torque allocation ensures vehicle stability and facilitates control over the vehicle’s stability.

## 4. Simulation Analysis

To comprehensively assess the effectiveness of the control strategy, two different working conditions were simulated using the proposed yaw stability controller described in Section 3. The effectiveness of four different control strategies, including the Linear–quadratic regulator optimized by the GA-PSO algorithm (referred to as GA-PSO-LQR), the traditional Linear–quadratic regulator (referred to as LQR), the traditional sliding mode controller (referred to as SMC), and the without additional yaw moment control (referred to as Without Control) were simulated and compared. The optimization parameters of the linear quadratic regulator were obtained through parameter optimization of GA-PSO. For Condition 1, the parameters were set to Q = diag[5.6849 × 10^4^, 7.5270 × 10^4^] and R = 10^−5^ and Q = diag[6.6397 × 10^4^, 9.1360 × 10^4^] and R = 10^−6^ for Condition 2. For the parameters of LQR and SMC, the optimal empirical values were Q = diag[10^4^, 10^4^] and R = 10^−5^. The SMC controller in this article adopted an exponential reaching law, where ε and k were the approaching law parameters ε = 0.01 and k = 50, respectively. The performance of the SMC and LQR controllers used in this article was similar to other controllers, but not better, as different calibrations can lead to different quantitative results. We strived to maximize the performance of each controller to ensure the rationality of simulation comparisons.

The main parameters of the vehicle are listed in Table 2. We used fitting or optimization techniques and identified the equivalent parameters of the monorail model by comparing data between the TruckSim model and the selected monorail model. This can be achieved by comparing the data of the monorail model with the Trucksim model at a given operating point or during actual driving. Optimization algorithms can minimize the error between actual observation data and monorail model simulation data to find the optimal equivalent parameter settings.

The Trucksim model uses a 2-axle electric truck (2A LCF Van). The turning part is Linear, 1/25 (Typical). The suspension part is a Typical 5.5 ton (12,000 lb) GAWR axle. The Trucksim uses the magic tire model, with specific parameters as follows: the front wheels are single tires, the wheels are dual tires, and the tires are radial tires with a load rating of 3000 kg. The power system is driven by four wheels hub motors, and the driving torque is input by the controller in Simulink.

### 4.1. Simulation Analysis of Serpentine Working Conditions

**Figure 4:**
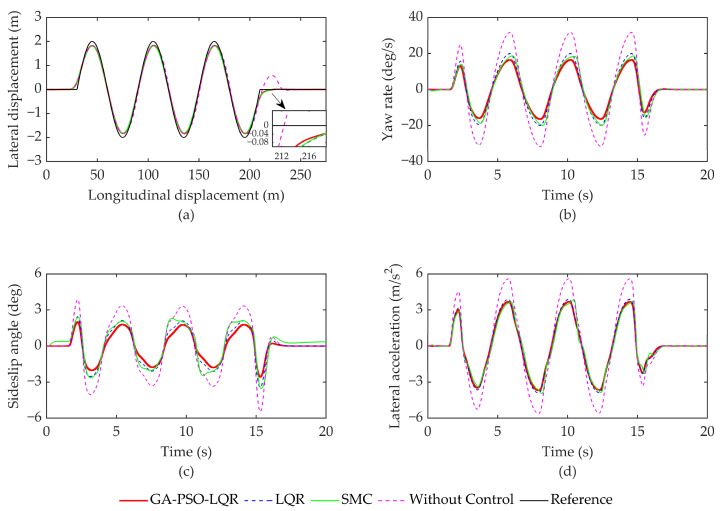
Simulation results of serpentine working conditions. (**a**) Track position; (**b**) Yaw rate; (**c**) Sideslip angle; and (**d**) Lateral acceleration.

Condition 1 entails simulating a serpentine maneuver at a speed of 50 km/h on a road surface with a friction coefficient of μ = 0.4. The simulation results are depicted in Figure 4. The specific evaluation index data can be found in Table 3. Figure 4a shows the trajectory position of the vehicle under four different control strategies, whereas Figure 4b shows the trend in the vehicle’s yaw rate change, Figure 4c shows the trend in the wheel sideslip angle change, and Figure 4d shows the trend in the vehicle’s lateral acceleration change.

As illustrated in Figure 4a, in the absence of control, the error significantly increases at locations with notable changes in path curvature. The other three control strategies, namely, LQR and SMC, can ensure that the vehicle follows the intended trajectory. However, the GA-PSO-LQR algorithm exhibits the least path deviation compared to the other strategies. Figure 4b illustrates that the yaw rate of the vehicle experiences significant changes Without Control. With the addition of the controller, the yaw rate can be controlled within 20 deg/s. The GA-PSO-LQR controller proposed in this study demonstrates yaw rate control effects 17.2%, 17.6%, and 48.1% better than SMC, LQR, and no control, respectively. Additionally, the root mean square (RMS) has been optimized by 14.9%, 17.5%, and 48.0%, respectively. The controller presented in this article ensures better stability during vehicle operation.

The response of the sideslip angle, as shown in Figure 4c, can be controlled within 4 degrees under the other three controllers compared to the Without Control scenario, thereby increasing the driving stability of the vehicle. In terms of the absolute maximum values, the controller proposed in this article has been optimized by 28.9%, 25.5%, and 53.1% compared to SMC, LQR, and Without Control, respectively. The RMS values have also been optimized by 26.2%, 18.8%, and 48.9%, respectively, for the same comparison. Lateral acceleration is an important indicator for evaluating the stability of vehicle driving. Based on the relevant data in Figure 4d and Table 3, after adding the yaw moment controller control, the lateral acceleration of the vehicle is effectively controlled within 4 m/s^2^, ensuring the stability of vehicle driving. Compared with SMC, LQR control, and Without Control, the GA-PSO-LQR controller proposed in this paper achieves optimizations of 6.4%, 5.4%, and 34.2% for the maximum absolute value of lateral acceleration and 4.8%, 9.1%, and 36.9% for RMS, respectively.

### 4.2. Simulation Double Lane Change Working Conditions

**Figure 5:**
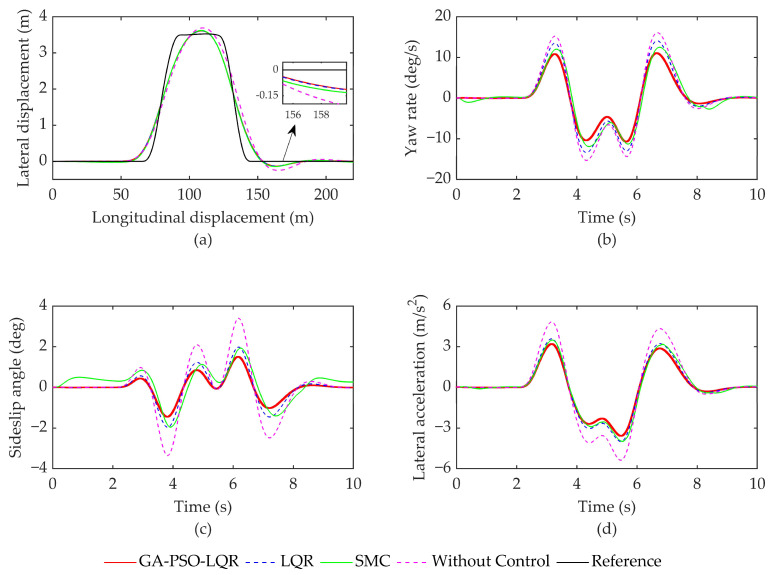
Simulation results of s double lane change working conditions. (**a**) Track position; (**b**) Yaw rate; (**c**) Sideslip angle; and (**d**) Lateral acceleration.

The simulation results for condition 2 are presented in Figure 5. Condition 2 refers to a double lane change simulation conducted at a speed of 80 km/h on a road surface with μ = 0.7. The relevant evaluation indicator data are recorded in Table 4.

From Figure 5a, it is evident that the addition of yaw moment control significantly reduces the lateral error compared to vehicles Without Control. Figure 5b demonstrates that the control strategy proposed in this paper enhances the effectiveness of yaw rate control compared to the other three strategies. Additionally, Figure 5c reveals that the uncontrolled vehicle exhibits a more rapid change in the centroid sideslip angle after 3 s, with an amplitude close to 4 degrees. The three control strategies GA-PSO-LQR, LQR, and SMC effectively suppress vehicle sideslip. On the other hand, the controller proposed in this paper can decrease the sideslip angle of the vehicle’s center of mass. The yaw rates of GA-PSO-LQR in this paper decreased by 11.6%, 21.6%, and 31.2% compared to SMC, LQR, and Without Control, respectively. Similarly, the sideslip angles decreased by 22.9%, 24.0%, and 55.7%, respectively, in terms of the absolute maximum value.

From the lateral acceleration data in Figure 5d and Table 4, it is evident that the vehicles without yaw moment control exhibit a lateral acceleration close to 5.4 m/s^2^, resulting in a significant reduction in vehicle driving stability. However, by implementing three controllers, the lateral acceleration of the vehicle can be effectively limited to within 4 m/s^2^. Comparing the results with SMC, LQR control, and Without Control, it is observed that the proposed GA-PSO-LQR controller optimizes 10.3%, 10.4%, and 33.4% of the maximum absolute values of lateral acceleration, respectively. Additionally, on the RMS measure, the GA-PSO-LQR controller achieves optimizations of 9.5%, 10.9%, and 35.9%, respectively.

### 4.3. Comparative Analysis of Torque Distribution

**Figure 6:**
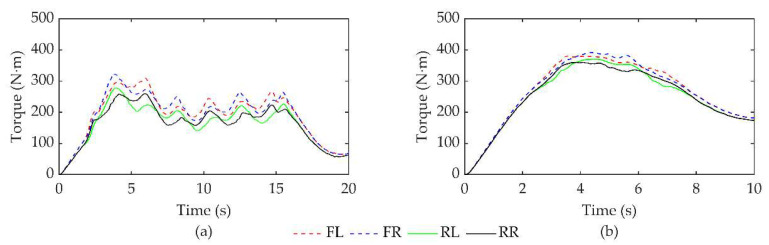
Torque of four wheels under two working conditions (GA-PSO-LQR controller). (**a**) Serpentine working condition. (**b**) Double lane change working condition.

Figure 6a,b shows the optimal torque distribution curves for all four wheels using the GA-PSO-LQR controller in two different working conditions: serpentine and double lane shifting. From the figures, it is evident that the torque applied to the front wheels is higher than that applied to the rear wheels due to variations in load distribution. Furthermore, by adjusting the torque of the vehicle’s left and right wheels, an improved stability can be achieved through optimized distribution. For each wheel, the torque exerted on the front wheel is consistently greater than that on the rear wheel, highlighting the effectiveness of the designed optimized allocation control strategy in fully utilizing tire adhesion on axles with larger loads and enhancing stability on axles with smaller loads.

**Figure 7:**
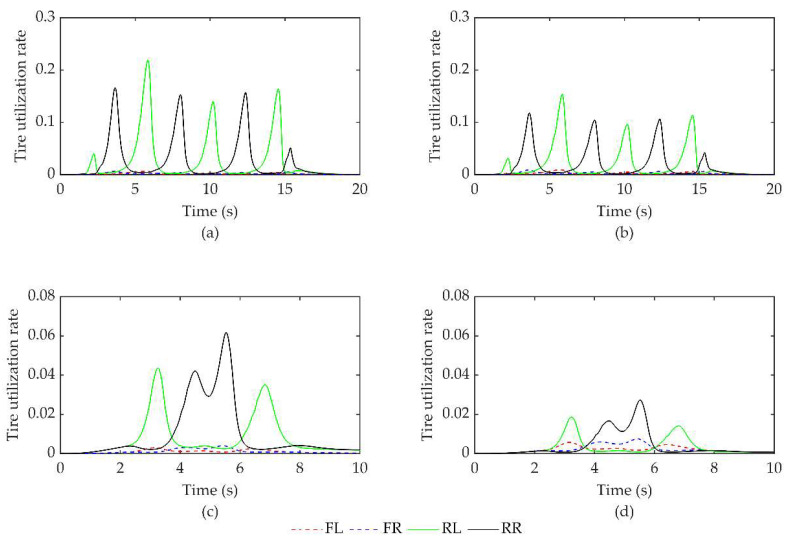
Tire utilization rate under two working conditions. (**a**) Equal distribution under serpentine working conditions. (**b**) Optimized distribution of serpentine working conditions. (**c**) Equal distribution of double lane change working conditions. (**d**) Optimized distribution of double lane change working conditions.

The effects of equal distribution and optimized distribution are compared and analyzed based on the controller discussed in this article. The stability is evaluated using the tire utilization rate as the index. The accurate tire utilization rate is calculated using the formula in Equation (24) for comparative analysis. The maximum tire utilization simulation data are recorded in Table 5. The tire utilization curves of different torque distribution methods under two working conditions are presented in Figure 7.

Figure 7a,b shows the tire utilization curves under two distribution methods for the serpentine working condition, whereas Figure 7c,d shows the curves under two distribution methods for the double lane shifting working condition. Referring to Table 5, it is evident that the optimized distribution reduces the maximum tire utilization by 29.4% and 55.8% under the respective working conditions compared to the equal distribution, thus improving the vehicle stability control margin.

## 5. Conclusions

This study aims to address the issue of yaw stability control in distributed drive electric trucks. The proposed framework introduces hierarchical control strategies. The upper controller determines the additional yaw moment using the LQR yaw moment controller, with the yaw rate error and center of mass sideslip angle as state variables. The weights of the LQR were optimized using GA-PSO. The lower layer torque distribution controller employs the Quadratic programming method to achieve optimal torque distribution in real-time, enhancing road adhesion and improving vehicle stability control. Four different control strategies were simulated and verified under serpentine and double lane change conditions. The results demonstrate that the proposed GA-PSO-LQR controller maintains a small tracking error while ensuring the intended driving trajectory. Moreover, when compared to SMC, LQR, and Without Control, the average yaw rate optimizations were 14.4%, 19.6%, and 42.15%, respectively, and the average sideslip angle reductions were 25.9%, 24.8%, and 52.3%, respectively. Additionally, the average lateral accelerations decreased by 8.4%, 7.9%, and 33.8%, respectively. These improvements significantly enhanced the yaw stability and driving safety of the vehicle. The influence of two different torque distribution methods on vehicle stability was also investigated. In both cases, the Quadratic programming-based distribution resulted in an average reduction in tire utilization by 42.6%, significantly improving the stability margin and driving stability of the vehicle. The effectiveness of the proposed control strategy has been successfully verified.

However, it should be noted that the parameter optimization of GA-PSO-LQR proposed in this study is still in an offline optimization state. Therefore, real-time optimization of LQR parameters under different working conditions and variable vehicle parameters is a topic that requires further investigation. Subsequently, actual vehicle experiments will be conducted to further verify the effectiveness of the control strategy.

## Figures and Tables

**Table 1:** Optimization results of weight matrix under two different operating conditions.

Condition	Q	R
1	[5.6849 × 10^4^, 7.5270 × 10^4^]	10^−5^
2	[6.6397 × 10^4^, 9.1360 × 10^4^]	10^−6^

**Table 2:** Main parameters of the vehicle.

Parameters/Units	Symbol	Value
Vehicle mass/kg	m	5760
Distance from the center of mass to the front axis/mm	a	1250
Distance from the center of mass to the rear axis/mm	b	3750
Moment of inertia/kg·m^2^	$I_{z}$	35,402.8
Front axle cornering stiffness/N/rad	$C_{f}$	322,450
Rear axle cornering stiffness/N/rad	$C_{r}$	330,030
Wheelbase of the front axle/mm	$d_{f}$	2030
Wheelbase of the rear axle/mm	$d_{r}$	1863
Height of the center of mass/mm	h	1175
Effective radius of wheel/mm	R	510

**Table 3:** Stability evaluation data for serpentine working conditions.

Performance Index	Yaw Rate (deg/s)		Sideslip Angle (deg)		Lateral Acceleration (m/s)	
	Maximum Value (abs)	RMS	Maximum Value (abs)	RMS	Maximum Value (abs)	RMS
Without Control	31.77	18.38	5.461	2.255	5.622	3.183
LQR	20.00	11.57	3.437	1.419	3.906	2.212
SMC	19.94	11.23	3.538	1.562	3.950	2.111
GA-PSO-LQR	16.48	9.549	2.562	1.152	3.697	2.010

**Table 4:** Stability evaluation data for double lane change working conditions.

Performance Index	Yaw Rate (deg/s)		Sideslip Angle (deg)		Lateral Acceleration (m/s)	
	Maximum Value (abs)	RMS	Maximum Value (abs)	RMS	Maximum Value (abs)	RMS
Without Control	16.07	7.736	3.396	1.334	5.368	2.517
LQR	14.09	6.779	1.980	0.7797	3.986	1.869
SMC	12.50	6.236	1.951	0.8233	3.981	1.841
GA-PSO-LQR	11.05	5.400	1.505	0.5723	3.572	1.666

**Table 5:** Optimization of tire utilization under two working conditions.

Working Conditions	Equal Distribution (Maximum)	Optimized Distribution (Maximum)	Optimized Proportion (Equal–Optimized)/Equal
Serpentine	0.2188	0.1537	29.4%
Double lane change	0.0618	0.0273	55.8%

## Data Availability

Not applicable.

## Abbreviations

**Abbreviations**	**Explanations**
LQR	Linear Quadratic Regulator
SMC	Sliding Mode Control
GA	Genetic Algorithm
PSO	Particle Swarm Optimization
GA-PSO	hybrid Genetic Algorithm and Particle Swarm Optimization algorithm
GA-PSO-LQR	Linear Quadratic Regulator optimized by hybrid Genetic Algorithm and Particle Swarm Optimization algorithm
Without Control	Without additional yaw moment control
